# *Pseudomonas aeruginosa* elastase causes transient disruption of tight junctions and downregulation of PAR-2 in human nasal epithelial cells

**DOI:** 10.1186/1465-9921-15-21

**Published:** 2014-02-18

**Authors:** Kazuaki Nomura, Kazufumi Obata, Takashi Keira, Ryo Miyata, Satoshi Hirakawa, Ken-ichi Takano, Takayuki Kohno, Norimasa Sawada, Tetsuo Himi, Takashi Kojima

**Affiliations:** 1 Departments of Otolaryngology, Sapporo Medical University School of Medicine, Sapporo 060-8556, Japan; 2 Departments of Pathology, Sapporo Medical University School of Medicine, Sapporo 060-8556, Japan; 3 Departments of Pediatrics, Sapporo Medical University School of Medicine, Sapporo 060-8556, Japan; 4 Department of Cell Science, Research Institute for Frontier Medicine, Sapporo Medical University School of Medicine, South-1, West-17, Chuo-ku, Sapporo 060-8556, Japan

**Keywords:** *Pseudomonas aeruginosa* elastase, Tight junctions, Barrier function, Human nasal epithelial cells, Signal transduction, PAR-2

## Abstract

**Background:**

*Pseudomonas aeruginosa* causes chronic respiratory disease, and the elastase enzyme that it produces increases the permeability of airway epithelial cells owing to the disruption of tight junctions. *P. aeruginosa* is also implicated in prolonged chronic rhinosinusitis. However, the effects of *P. aeruginosa* elastase (PE) against the barrier formed by human nasal epithelial cells (HNECs) remain unknown.

**Methods:**

To investigate the mechanisms involved in the disruption of tight junctions by PE in HNECs, primary cultures of HNECs transfected with human telomerase reverse transcriptase (hTERT-HNECs) were used. The hTERT-HNECs were pretreated with inhibitors of various signal transduction pathways, PKC, MAPK, p38MAPK, PI3K, JNK, NF-κB, EGF receptor, proteasome, COX1 and COX2 before treatment with PE. Some cells were pretreated with siRNA and agonist of protease activated receptor-2 (PAR-2) before treatment with PE. Expression and structures of tight junctions were determined by Western blotting, real-time PCR, immunostaining and freeze-fracture. Transepithelial electrical resistance (TER) was examined as the epithelial barrier function.

**Results:**

PE treatment transiently disrupted the epithelial barrier and downregulated the transmembrane proteins claudin-1 and -4, occludin, and tricellulin, but not the scaffold PDZ-expression proteins ZO-1 and -2 and adherens junction proteins E-cadherin and β-catenin. The transient downregulation of tight junction proteins was controlled via distinct signal transduction pathways such as the PKC, MAPK, PI3K, p38 MAPK, JNK, COX-1 and -2, and NF-κB pathways. Furthermore, treatment with PE transiently decreased PAR-2 expression, which also regulated the expression of the tight junction proteins. Treatment with a PAR-2 agonist prevented the downregulation of the tight junction proteins after PE treatment in HNECs.

**Conclusions:**

PE transiently disrupts tight junctions in HNECs and downregulates PAR-2. The transient disruption of tight junctions by PE might occur repeatedly during chronic rhinosinusitis.

## Introduction

*Pseudomonas aeruginosa* (*P. aeruginosa*) is a virulent Gram-negative bacterium that causes aggressive infections in patients compromised by pre-existing respiratory disease such as cystic fibrosis and diffuse panbronchiolitis [1,2]. *P. aeruginosa* is also associated with prolonged chronic rhinosinusitis (CRS) [3].

*P. aeruginosa* secretes several virulence factors such as exotoxin A, exoenzyme S, pyocyanin, and elastase, which play an important role in pathogenesis [4,5]. *P. aeruginosa* elastase (PE) increases paracellular permeability in lung epithelial cells via mechanisms involving tight junction disruption and cytoskeletal reorganization [6]. PE affects epithelial cells via multiple mediators of signaling including activation of PKC, EGFR, ERK1/2, NF-κB, urokinase/uPAR, and protease activated receptor-2 (PAR-2) [1,2,7-11]. PKC signaling is involved in PE-induced epithelial barrier disruption via tight junction translocation and cytoskeletal reorganization in the human bronchial adenocarcinoma cell line Calu-3 [2].

PE disables PAR-2 in respiratory epithelial cells [1]. Protease-activated receptors (PARs) are G protein-coupled receptors with seven transmembrane domains, which are cleaved at an activation site within the N-terminal exodomain by a variety of proteases [1]. Four PARs (PAR-1, -2, -3, and -4) have been identified and are widely expressed by cells in blood vessels, connective tissue, leukocytes, epithelium, and many airway cells [12]. PAR-2 is expressed in airway epithelium, and its activation initiates multiple effects including enhanced airway inflammation and reactivity [13]. Upregulation of PAR-2 is observed in the respiratory epithelium of patients with asthma and chronic rhinosinusitis [14,15]. PAR-2 activation also affects the airway epithelial barrier [16]. However, details of the mechanistic effects of PE against the epithelial barrier via PAR-2 remain unknown.

Airway epithelium of human nasal mucosa acts as a physical barrier that protects against inhaled substances and pathogens because of its tight junctions, the most apical intercellular junctions [17-19]. Tight junctions are formed by not only the integral membrane proteins claudins, occludin, tricellulin, and junctional adhesion molecules (JAMs), but also by many peripheral membrane proteins, including the scaffold PDZ-expression proteins zonula occludens (ZO) and non-PDZ-expressing proteins [20-23]. We previously reported that, in HNECs *in vivo*, the tight junction molecules occludin, tricellulin, JAM-A, claudin-1, -4, -7, -8, -12, -13, and -14, and ZO-1 and -2 were detected together with well-developed tight junction strands [17,24,25]. The tight junctions and the well-developed barrier function in primary *in vitro* cultures of HNECs transfected with human telomerase reverse transcriptase (hTERT-HNECs) were very similar to those observed in HNECs *in vivo*[24-27]. Furthermore, in the *in vitro* HNECs, tight junction molecules and barrier function are upregulated by various stimuli via distinct signal transduction pathways [25].

In the present study, to investigate the effects of elastase on the tight junction barrier of HNECs, hTERT-HNECs were treated with PE. Treatment with PE transiently disrupted the epithelial barrier and downregulated the transmembrane proteins claudin-1 and -4, occludin, and tricellulin but not the scaffold PDZ-expression proteins ZO-1 and -2 and adherens junction proteins E-cadherin and β-catenin. Downregulation of tight junction proteins because of PE treatment was mediated via distinct signal transduction pathways. Furthermore, treatment with PE transiently decreased PAR-2 expression, which partially regulated the expression of the tight junction proteins. A PAR-2 agonist prevented the downregulation of tight junction proteins after PE treatment in HNECs.

## Materials and methods

### Reagents

A pan-PKC inhibitor (GF109203X), MEK1/2 inhibitor (U0126), p38 MAPK inhibitor (SB203580), and PI3K inhibitor (LY294002) were purchased from Calbiochem-Novabiochem Corporation (San Diego, CA). JNK inhibitor (SP600125) and NF-κB inhibitor (IMD-0354) were purchased from Sigma-Aldrich (St. Louis, MO). Epidermal growth factor (EGF) receptor inhibitor (AG1478) was purchased from Calbiochem-Novabiochem Corporation (San Diego, CA). Proteasome inhibitor (MG132), the COX1 inhibitor (FR122047), and COX2 inhibitor were purchased from Calbiochem Novabiochem Corporation (San Diego, CA). *Pseudomonas aeruginosa* elastase and neutrophil elastase were purchased from Elastin Products Company, Inc. (Owensville, USA). Protease activated receptor 2 (PAR-2) agonist (*SLIGKV*-NH2) was purchased from R&D Systems, Inc. (Minneapolis, MN). Alexa 488 (green)- and Alexa 594 (red)-conjugated anti-mouse and anti-rabbit IgG antibodies were purchased from Invitrogen. HRP-conjugated polyclonal goat anti-rabbit immunoglobulins were purchased from Dako A/S (Glostrup, Denmark). The ECL Western blotting system was obtained from GE Healthcare UK, Ltd. (Buckinghamshire, UK).

### Cell culture and treatments

The cultured HNECs were derived from the mucosal tissues of patients who underwent inferior turbinectomy at the Sapporo Hospital of Hokkaido Railway Company and the KKR Sapporo Medical Center Tonan Hospital. Informed consent was obtained from all patients and this study was approved by the ethical committees of Sapporo Medical University, the Sapporo Hospital of Hokkaido Railway Company, and the KKR Sapporo Medical Center Tonan Hospital.

The procedures for primary culture of human nasal epithelial cells were as reported previously [26]. Primary cultured HNECs were transfected with the catalytic component of telomerase, the human catalytic subunit of the telomerase reverse transcriptase (hTERT) gene as described previously [26]. The cells were plated on 35-mm or 60-mm culture dishes (Corning Glass Works, Corning, NY, USA), which were coated with rat tail collagen (500 μg of dried tendon/ml 0.1% acetic acid). The cells were cultured in serum-free bronchial epithelial cell basal medium (BEBM, Lonza Walkersville, Inc.; Walkersville, MD, USA) supplemented with bovine pituitary extract (1% v/v), 5 μg/ml insulin, 0.5 μg/ml hydrocortisone, 50 μg/ml gentamycin, 50 μg/ml amphotericin B, 0.1 ng/ml retinoic acid, 10 μg/ml transferrin, 6.5 μg/ml triiodothyronine, 0.5 μg/ml epinephrine, 0.5 ng/ml epidermal growth factor (Lonza Walkersville, Inc.), 100 U/ml penicillin and 100 μg/ml streptomycin (Sigma-Aldrich) and incubated in a humidifier, 5% CO2:95% air incubator at 37°C. This experiment used cells in the second and third passage. The hTERT-HNECs were treated with 0.1 U (a unit of 3.83 μg/ml) *Pseudomonas aeruginosa* elastase (PE) or 0.01 U (a unit of 1.25 μg/ml) neutrophil elastase (NE). Some cells were pretreated with or without inhibitors of pan-PKC, MEK1/2, p38MAPK, PI3K, JNK, NF-κB, EGF receptor, proteasome, COX1, COX2 and PAR-2 agonist 30 min before treatment with 0.1 U PE. The concentrations of the various inhibitors were used following our previous reports [28,29].

### Transfection with small interfering RNA (siRNA)

siRNA duplex oligonucleotides against human PAR 2 (sc-36188) were synthesized by Santa Cruz Biotechnology, inc. (Santa Cruz, CA). The hTERT-transfected HNECs at 24 h after plating were transfected with 100 nM siRNA of PAR-2 using Lipofectamine™ RNAiMAX Reagent (Invitrogen). Some cells were treated with 0.1 U PE after transfection with 100 nM siRNS of PAR-2 for 48 h.

### RNA isolation, RT-PCR, and real-time RT-PCR analysis

Total RNA was extracted and purified from hTERT-transfected HNECs using TRIzol reagent (Invitrogen). One microgram of total RNA was reverse transcribed into cDNA using a mixture of oligo(dT) and Superscript II RTase using the recommended conditions (Invitrogen). Each cDNA synthesis was performed in a total volume of 20 μl for 50 min at 42°C and terminated by incubation for 15 min at 70°C. PCR containing 100 pM primer pairs and 1.0 μl of the 20 μl total RT reaction was performed in 20 μl of 10 mM Tris–HCl (pH 8.3), 50 mM KCl, 1.5 mM MgCl2, 0.4 mM dNTPs, and 0.5 U of Taq DNA polymerase (Takara Bio, Inc.; Shiga, Japan), employing 25, 30, or 35 cycles with cycle times of 15 s at 96°C, 30 s at 55°C, and 60 s at 72°C. The final elongation step was 7 min at 72°C. Nine microliters of the 20 μl total PCR reaction was analyzed by gel electrophoresis with 2% agarose after staining with ethidium bromide. To provide a quantitative control for reaction efficiency, PCR reactions were performed with primers coding for the housekeeping gene glyceraldehyde-3-phosphate dehydrogenase (G3PDH). Primers used to detect G3PDH, occludin, tricellulin, claudin-1, -4, -7, PAR-1, and -2 are indicated in Table 1.

**Table 1:** Primers for RT-PCR

**Gene**	**Forward primer**	**Reverse primer**	**Product size (bp)**
G3PDH	ACCACAGTCCATGCCATCAC	TCCACCACCCTGTTGCTGTA	452
PAR-1	GGATATTTGACCAGCTCCTGG	AGATGGCCAGACAAGTGAAGG	400
PAR-2	CTGCATCTGTCCTCACTGGAA	ATTGCCAGGGAGATGCCAATG	400
Claudin-1	GCTGCTGGGTTTCATCCTG	CACATAGTCTTTCCCACTAGAAG	619
Claudin-4	AGCCTTCCAGGTCCTCAACT	AGCAGCGAGTAGAAG	249
Claudin-7	AGGCATAATTTTCATCGTGG	GAGTTGGACTTAGGGTAAGAGCG	252
Occludin	TCAGGGAATATCCACCTATCACTTCAG	CATCAGCAGCAGCCATGTACTCTTCAC	189
Tricellulin	AGGCAGCTCGGAGACATAGA	TCACAGGGTATTTTGCCACA	200

Real-time PCR detection was performed using a TaqMan Gene Expression Assay kit with a StepOnePlus™ real-time PCR system (Applied Biosystems; Foster City, CA, USA). The amount of 18S ribosomal RNA (rRNA) (Hs99999901) in each sample was used to standardize the quantity of the following mRNAs: tricellulin (Hs00930631), claudin-1 (Hs00221623), claudin-4 (Hs00533616), claudin-7 (Hs00154575), occludin (Hs00170162).

The relative mRNA expression levels between the control and treated samples were calculated by the difference of the threshold cycle (comparative C_T_ [∆∆C_T_] method) and presented as the average of triplicate experiments with a 95% confidence interval.

### Western blot analysis

The hTERT-transfected HNECs were scraped from a 60 mm dish containing 300 μl of buffer (1 mM NaHCO3 and 2 mM phenylmethylsulfonyl fluoride), collected in microcentrifuge tubes, and then sonicated for 10 s. The protein concentrations of the samples were determined using a BCA protein assay reagent kit (Pierce Chemical Co.; Rockford, IL, USA). Aliquots of 15 μl of protein/lane for each sample were separated by electrophoresis in 5–20% SDS polyacrylamide gels (Daiichi Pure Chemicals Co.; Tokyo, Japan), and electrophoretic transfer to a nitrocellulose membrane (Immobilon; Millipore Co.; Bedford, UK) was performed.

The membrane was saturated for 30 min at room temperature with blocking buffer (25 mM Tris, pH 8.0, 125 mM NaCl, 0.1% Tween 20, and 4% skim milk) and incubated with anti-claudin-1, -4, and -7 anti-occludin, anti-tricellulin, anti-ZO-1, and -2, anti-β-catenin, anti-E-cadherin, anti-actin and anti-PAR-2 antibodies (Table 2) at room temperature for 1 h. The membrane was incubated with HRP-conjugated anti-mouse and anti-rabbit IgG antibodies at room temperature for 1 h. The immunoreactive bands were detected using an ECL Western blotting system.

**Table 2:** Antibodies

**Antibody**	**Type**	**Dilution**		**Company**
		**IS**	**WB**	
claudin-1	pAb	1:100	1:1000	Zymed Laboratories (San Francisco, CA)
claudin-4	pAb		1:1000	Zymed Laboratories (San Francisco, CA)
claudin-7	pAb		1:1000	Zymed Laboratories (San Francisco, CA)
occludin	pAb	1:100	1:1000	Zymed Laboratories (San Francisco, CA)
tricellulin	pAb		1:1000	Zymed Laboratories (San Francisco, CA)
ZO-1	pAb		1:1000	Zymed Laboratories (San Francisco, CA)
ZO-2	pAb		1:1000	Zymed Laboratories (San Francisco, CA)
actin	pAb		1:1000	Sigma-Aldrich (St. Louis, MO)
E-cadherin	mAb (36)		1:2000	BD Biosciences (San Jose, CA)
β-catenin	pAb		1:1000	Zymed Laboratories (San Francisco, CA)

pAb; rabbit polyclonal antibody, mAb; mouse monoclonal antibody, IS; immunostaining, WB; Western blotting.

### Immunocytochemical staining

The hTERT-transfected HNECs grown in 35-mm glass-coated wells (Iwaki, Chiba, Japan), were fixed with cold acetone and ethanol (1:1) at –20°C for 10 min. After rinsing in PBS, the cells were incubated with anti-occludin and anti-claudin-1 antibodies (Table 2) at room temperature for 1 h. Alexa Fluor 488 (green)-conjugated anti-rabbit IgG and Alexa Fluor 592 (red)-conjugated anti-mouse IgG (Invitrogen) were used as secondary antibodies. The specimens were examined using a confocal laser scanning microscope (LSM510; Carl Zeiss, Jena, Germany).

### Freeze-fracture analysis

For the freeze-fracture technique, the cells were immersed in 40% glycerin solution after fixation in 2.5% glutaraldehyde/0.1 M phosphate-buffered saline (PBS). The specimens were fractured at –150°C to –160°C in a JFD-7000 freeze-fracture device (JEOL, Ltd., Tokyo, Japan) and replicated by deposition of platinum/carbon from an electron beam gun positioned at a 45° angle followed by carbon applied from overhead. Replicas were examined at 100kV with a JEM transmission electron microscope (JEOL Ltd., Tokyo, Japan).

### Continuous online measurements of transepithelial electrical resistance (TER)

Cells were cultured to confluence on the inner chambers of 12-mm Transwell 0.4-μm pore-size filters (Corning Life Science). Transepithelial electrical resistance (TER) was monitored using a cellZscope (nanoAnalytics, Germany), a computer controlled automated multi-well device (12 wells). The values are expressed in standard units of ohms per square centimeter and presented as the mean ± SD of triplicate experiments.

### Data analysis

Signals were quantified using Scion Image Beta 4.02 Win (Scion Co.; Frederick, MA). Each set of results shown is representative of at least three separate experiments. Results are given as means ± SEM. Differences between groups were tested by ANOVA followed by a *post-hoc* test and an unpaired two-tailed Student’s *t* test and considered to be significant when p < 0.05.

## Results

### *P. aeruginosa* elastase (PE) transiently reduces the expression of transmembrane proteins in the tight junctions in HNECs

**Figure 1:**
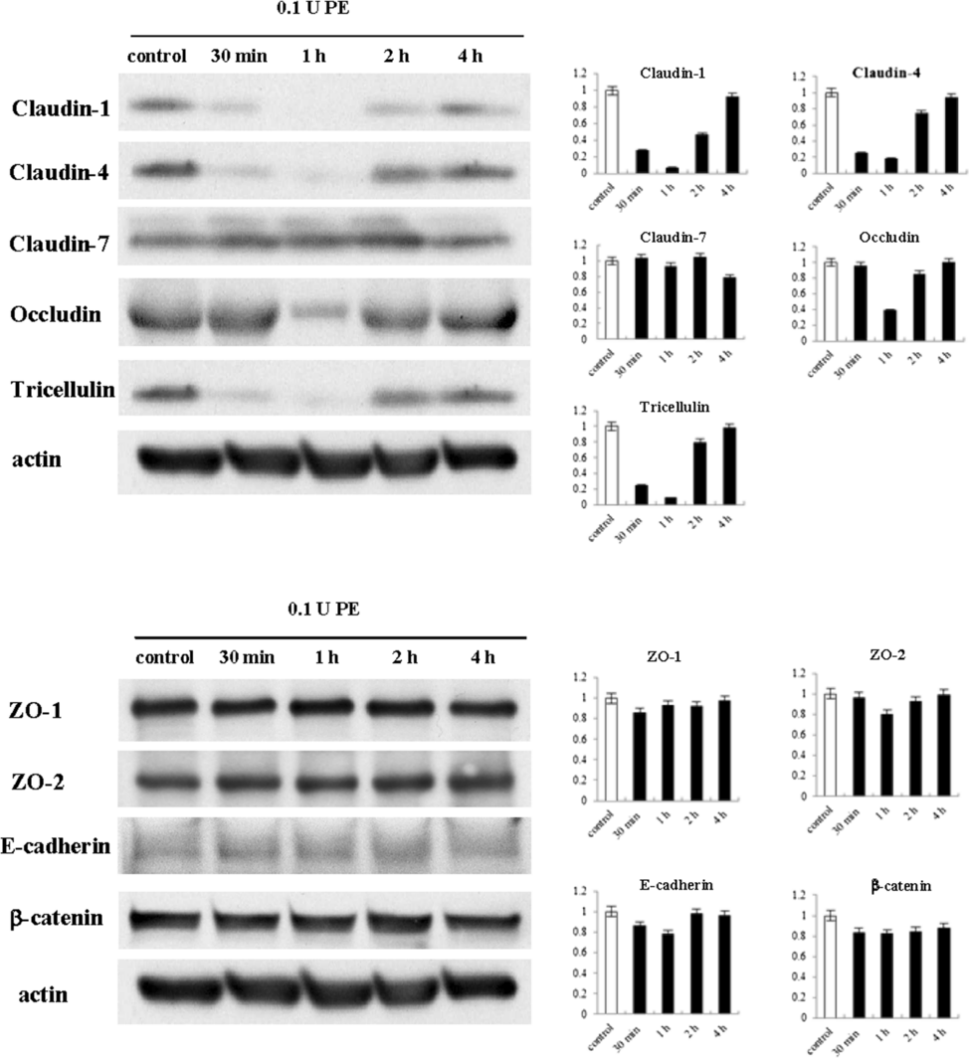
**Western blotting for tight junction and adherens junction proteins in hTERT-transfected HNECs after treatment with 0.1 U *Pseudomonas aeruginosa *elastase.** The corresponding expression levels are shown as bar graphs. PE: *Pseudomonas aeruginosa* elastase.

**Figure 2:**
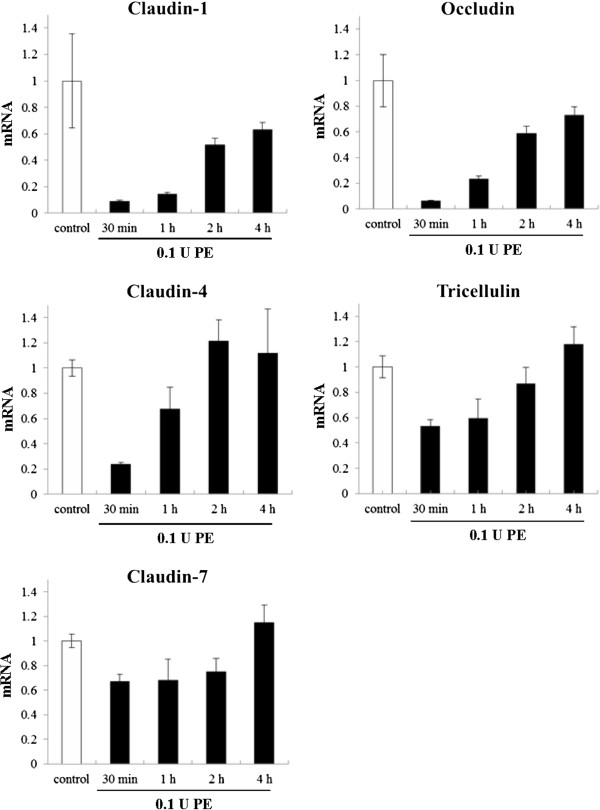
**Real-time PCR analysis of claudin-1, -4, and -7, occludin, and tricellulin mRNA in hTERT-transfected HNECs after treatment with 0.1 U *Pseudomonas aeruginosa *elastase.** PE: *Pseudomonas aeruginosa* elastase.

To investigate whether *Pseudomonas aeruginosa* elastase (PE) affects the protein and mRNAs expression of tight junction and adherens junction molecules in HNECs, hTERT-HNECs were treated with 0.1 U PE for 30 min, 1 h, 2 h, and 4 h. Western blots showed that claudin-1, -4, and tricellulin protein levels decreased at 30 min but were restored at 2 h, whereas occludin protein was transiently reduced at 1 h (Figure 1). No changes in claudin-7, ZO-1, ZO-2, E-cadherin, and β-catenin protein levels were observed post-treatment (Figure 1). The mRNA levels of claudin-1, -4, occludin, and tricellulin decreased at 30 min and were restored at 2 h, whereas claudin-7 mRNA level was slightly reduced from 30 min until 2 h (Figure 2).

Furthermore, we investigated the effects of neutrophil elastase (NE) on the expression of tight junction and adherens junction molecules in HNECs, to compare the effects of PE. When hTERT-HNECs were treated with 0.01 U NE for 30 min, 1 h, 2 h, and 4 h, claudin-1, occludin, and tricellulin protein levels were transiently reduced 30 min post-treatment with NE, while no changes in claudin-4, -7, ZO-1, ZO-2, E-cadherin and β-catenin protein levels were observed (Additional file 1). NE did not affect mRNA levels of claudin-1, occludin, and tricellulin (Additional file 1).

### PE affects the distribution of transmembrane tight junction proteins and the formation of tight junction strands in HNECs

**Figure 3:**
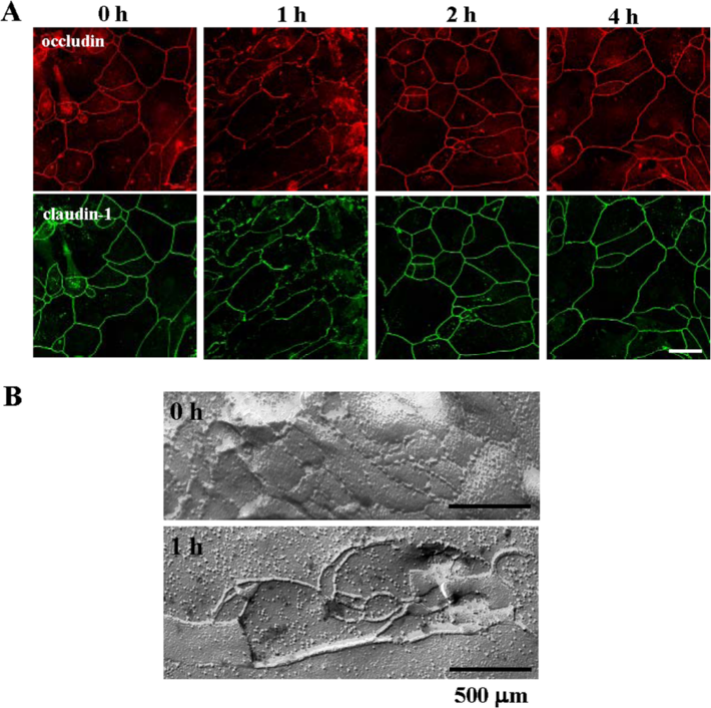
**Immunocytochemical and transmission electron microscopic findings. (A)** Immunocytochemical staining of occludin and claudin-1 and **(B)** Freeze-Fracture replicas in hTERT-transfected HNECs after treatment with 0.1 U *Pseudomonas aeruginosa* elastase. Bars: A=20 μm, B=500 μm.

We investigated changes in the distribution of tight junction proteins in hTERT-HNECs 1 h, 2 h, and 4 h post-treatment with 0.1 U PE. In the control cells (0 h), strong immunoreactivity of occludin and claudin-1 was observed at the membranes (Figure 3A). The immunoreactivities of occludin and claudin-1, in part, disappeared at the borders of some cells 1 h after treatment with PE and were then recovered 2 h post-treatment (Figure 3A).

Furthermore, we performed freeze-fracture analysis to investigate changes in the tight junction strands in hTERT-HNECs 1 h after treatment with 0.1 U of PE. In the control cells, a network composed of several continuous tight junction strands was observed (Figure 3B). The cells treated with PE exhibited a reduced number of tight junction strands, which were partially disrupted (Figure 3B).

### PE transiently reduces the tight junction barrier function of HNECs

**Figure 4:**
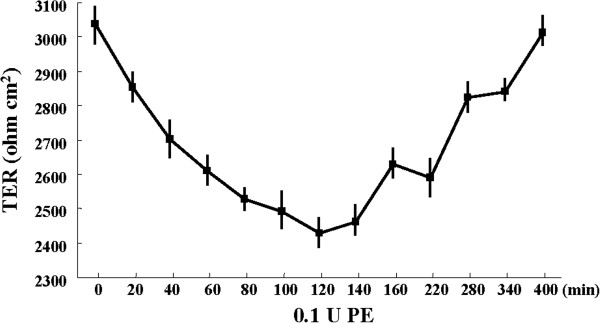
**Barrier function measured by TER in hTERT-transfected HNECs after treatment with 0.1 U *Pseudomonas aeruginosa *elastase.** PE: *Pseudomonas aeruginosa* elastase.

To investigate the effects of PE on the tight junction barrier function of HNECs, hTERT-HNECs were treated with 0.1 U PE and then examined with continuous online measurements of transepithelial electric resistance (TER) by using a cellZscope. As shown in Figure 4, the TER was continuously decreased from 20 min to 120 min after PE-treatment, and continuously increased from 140 min to 400 min, recovering to the level prior to PE-treatment (Figure 4).

### The reduction of transmembrane tight junction proteins by PE is regulated via distinct signaling pathway

**Figure 5:**
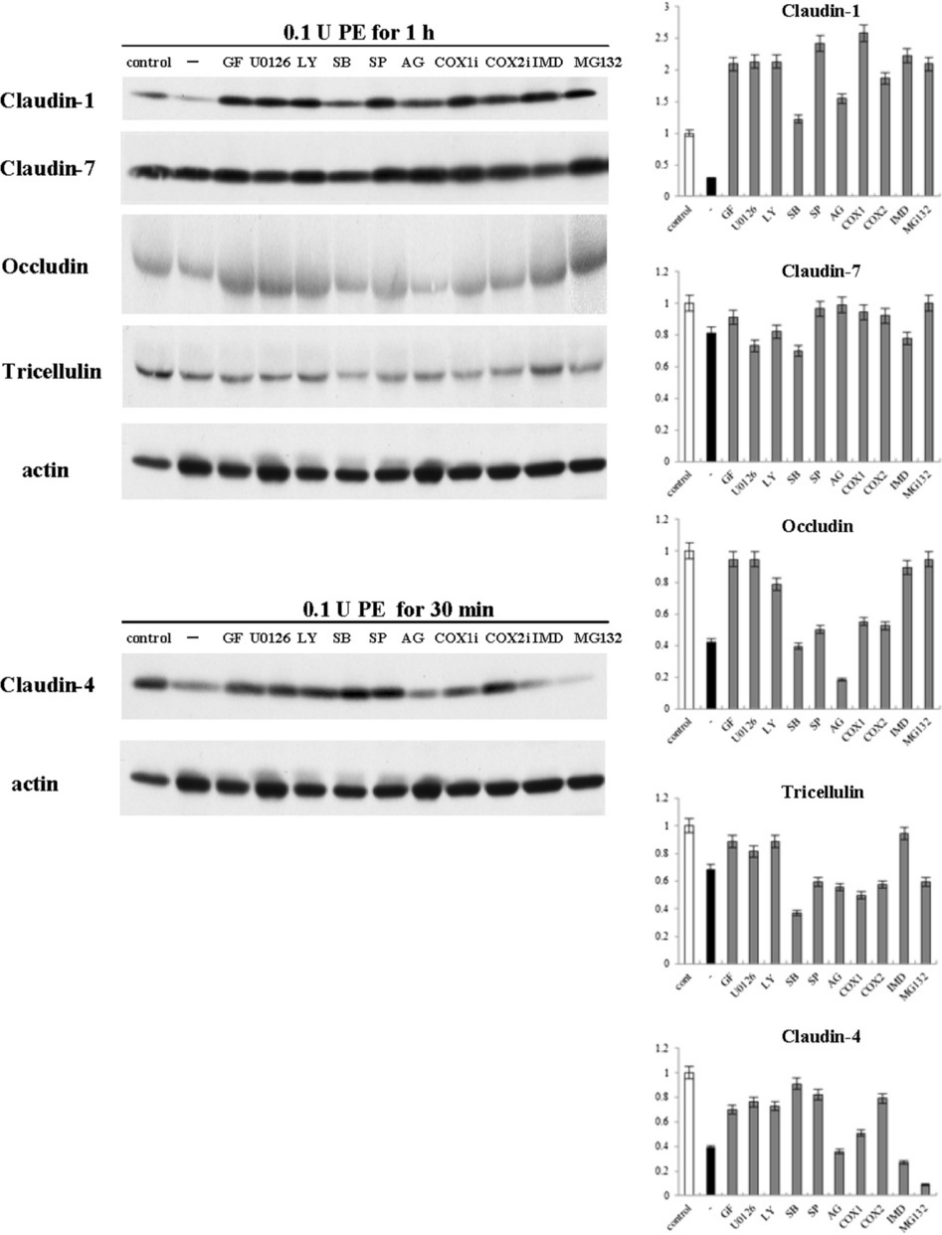
**Western blotting analysis.** Western blotting for tight junction proteins in hTERT-transfected HNECs pretreatment with pan-PKC inhibitor (GF109203X), MEK1/2 inhibitor (U0126), PI3K inhibitor (LY294002), p38 MAPK inhibitor (SB203580), JNK inhibitor (SP600125), epidermal growth factor (EGF) receptor inhibitor (AG1478), COX1 inhibitor (FR122047), and COX2 inhibitor, NF-κB inhibitor (IMD-0354), and Proteasome inhibitor (MG132) before treatment with 0.1 U *Pseudomonas aeruginosa* elastase for 30 min or 1 h. The corresponding expression levels are shown as bar graphs. PE: *Pseudomonas aeruginosa* elastase.

It is reported that PE disrupts tight junctions via a PKC pathway (2). To investigate which signal transduction pathways affected the reduction of transmembrane tight junction proteins in HNECs after treatment with PE, hTERT-HNECs were pretreated with inhibitors of pan-PKC (GF109203X), MEK1/2 (U0126), PI3K (LY294002), p38 MAPK (SB203580), JNK (SP600125), EGFR (AG1478), COX1 (FR122047), COX2, NF-κB (IMD-0354), and Proteasome (MG132) at each 10 μg/ml 30 min before treatment with 0.1 U PE for 30 min or 1 h. The reduction of claudin-1 and occludin at 1 h after treatment with PE was prevented by GF109203X, U0126, LY294002, SP600125, inhibitors of COX1 and COX2, IMD-0354 and MG132 (Figure 5). The reduction of tricellulin at 1 h after treatment with PE was prevented by GF109203X, U0126, LY294002, and IMD-0354 (Figure 5). GF109203X, U0126, LY294002, SB203580, SP600125, and inhibitors of COX1 and COX2 inhibited claudin-4 reduction 30 min post PE treatment (Figure 5). No change of all tight junction proteins was observed at the concentrations of the various inhibitors without PE (Additional file 2).

### PE reduces PAR-2 expression in HNECs

**Figure 6:**
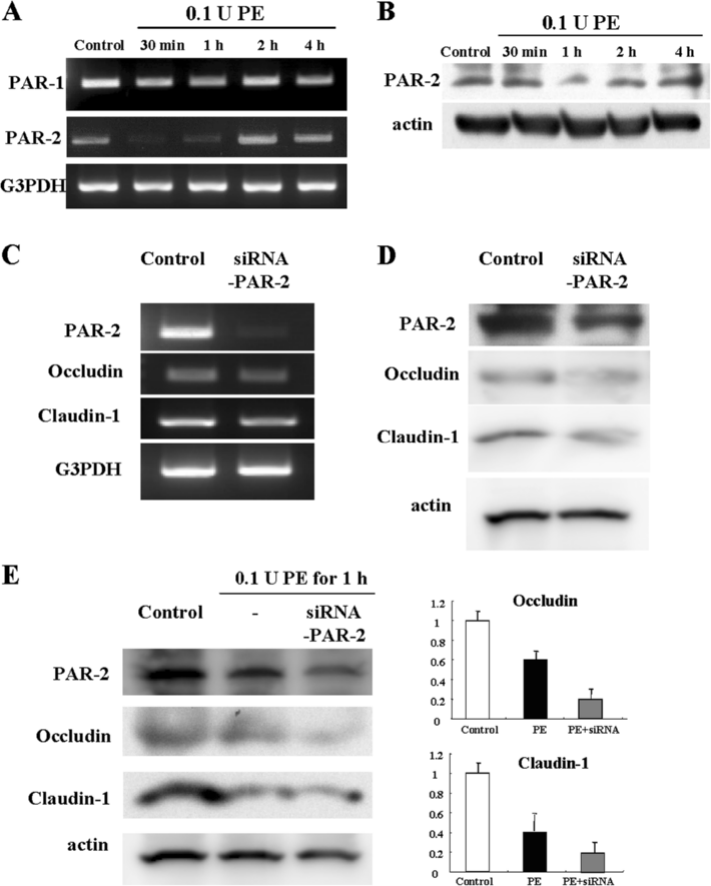
**RT-PCR and Western blotting analyses. (A)** RT-PCR for PAR-1 and -2 and **(B)** Western blotting for PAR-2 in hTERT-transfected HNECs after treatment with 0.1 U *Pseudomonas aeruginosa* elastase. **(C)** RT-PCR and **(D)** Western blotting for PAR-2, occludin, and claudin-1 in hTERT-transfected HNECs after treatment with siRNA of PAR-2 for 48 h. **(E)** Western blotting for PAR-2, occludin, and claudin-1 in hTERT-transfected HNECs pretreated with PAR-2 siRNA before treatment with 0.1 U *Pseudomonas aeruginosa* elastase for 1 h. The corresponding expression levels are shown as bar graphs. PE: *Pseudomonas aeruginosa* elastase.

PE disables PAR-2 in airway epithelial cells A549 and 16 HBE cells (1). To investigate whether PE affects PAR-2 expression in HNECs, mRNA and protein in hTERT-HNECs 30 min, 1 h, 2 h, and 4 h after treatment with 0.1 U PE were examined by RT-PCR and Western blotting. PAR-2 but not PAR-1 mRNA was markedly decreased at 30 min and was restored at 2 h (Figure 6A). PAR-2 protein was transiently reduced 1 h after treatment (Figure 6B).

### Knockdown of PAR-2 downregulates transmembrane tight junction proteins in HNECs with or without treatment with PE

We investigated whether PAR-2 expression affects transmembrane tight junction proteins in HNECs with or without treatment with PE. Occludin and claudin-1 mRNA and protein levels in hTERT-HNECs without PE-treatment were reduced by the knockdown of PAR-2 using siRNA (Figure 6C and D). The occludin and claudin-1 protein levels in hTERT-HNECs 1 h post treatment with 0.1 U PE decreased after the knockdown of PAR-2 (Figure 6E).

### The downregulation of transmembrane tight junction proteins by PE treatment is prevented by PAR-2 agonist

**Figure 7:**
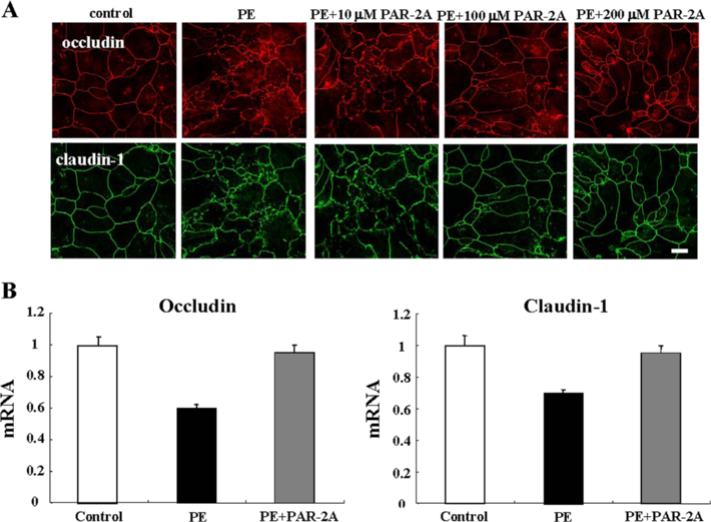
**Immunocytochemistrical finding and Real-time PCR analysis. (A)** Immunocytochemistrical staining for occludin and claudin-1 and occludin in hTERT-transfected HNECs pretreated with 10–200 μM PAR-2 agonist before treatment with 0.1 U *Pseudomonas aeruginosa* elastase for 1 h. Bar: 10 μm. **(B)** Real-time PCR for occludin and claudin-1 mRNAs in hTERT-transfected HNECs pretreated with 100 μM PAR-2 agonist before treatment with 0.1 U *Pseudomonas aeruginosa* elastase for 1 h. PE: *Pseudomonas aeruginosa* elastase. PAR-2A: PAR-2 agonist.

We investigated whether PAR-2 agonist prevents the reduction of transmembrane tight junction proteins caused by PE treatment in HNECs. When hTERT-HNECs were pretreated with 10–200 μM PAR-2 agonist 30 min before treatment with 0.1 U PE for 1 h, disruption of occludin and claudin-1 at the membranes after PE treatment was prevented in cells that were treated with PAR-2 agonist concentrations of 100 μM or more (Figure 7A). The downregulation of occludin and claudin-1 mRNAs by PE was prevented by treatment with 100 μM of PAR-2 agonist (Figure 7B).

## Discussion

In this study, we first found that PE transiently disrupted the epithelial barrier of HNECs by the downregulation of transmembrane tight junction proteins via distinct signal transduction pathways. Furthermore, PE decreased PAR-2 expression, which plays a crucial role in the maintenance of tight junction proteins in HNECs.

PE increases paracellular permeability in lung epithelial cells by causing tight junction disruption and cytoskeletal reorganization [6]. Furthermore, PE decreases epithelial barrier function in a time-dependent manner in the human bronchial adenocarcinoma cell line Calu-3 by reduced localization of occludin and ZO-1 in the membrane fraction [2]. In these airway epithelial cells, the barrier function is not recovered after treatment with PE. In the present study, treatment with PE transiently decreased the epithelial barrier of HNECs together with downregulation of the transmembrane proteins claudin-1 and -4, occludin, and tricellulin but not the scaffold PDZ-expression proteins ZO-1 and -2 and adherens junction proteins E-cadherin and β-catenin. Furthermore, reduced localization of occludin and claudin-1 and the disruption of tight junction structure were observed following PE treatment. Nevertheless the expression of claudin-1 and occludin by treatment with PE was markedly reduced at the level of mRNA and protein compared to the control, the immunostaining of these two proteins did not represent the dramatic reduction. In the present study using HNECs, it is possible that PE may strongly affect the synthesis of the tight junction proteins rather than the localization, although the detailed mechanisms are unclear. Treatment with NE also transiently decreased claudin-1, occludin, and tricellulin protein levels in HNECs. The sensitivity to PE in HNECs and other airway epithelial cells is different. PE, as a thermolysin-like metalloproteinase [30], may more strongly degrade the extracellular loops of transmembrane proteins in HNECs than other airway epithelial cells.

The tight junction proteins are regulated by various cytokines and growth factors via distinct signal transduction pathways [23,31]. In HNECs *in vitro*, tight junction proteins and the barrier function are also regulated by various stimuli via distinct signal transduction pathways [25]. On the other hand, PE affects the epithelial cells via multiple mediators of signaling, including activation of PKC, EGFR, Erk1/2, NF-κB, urokinase/uPAR and protease activated receptor-2 (PAR-2) [1,2,7-11]. PKC signaling is involved during PE-induced epithelial barrier disruption via tight junction translocation and cytoskeletal reorganization in the human bronchial adenocarcinoma cell line Calu-3 [2]. In the present study of HNECs, the transient downregulation of the transmembrane tight junction proteins by treatment with PE was controlled via distinct signal transduction pathways such as PKC, MEK1/2, PI3K, p38 MAPK, JNK, COX-1, -2 and NF-κB. Furthermore, there are, in part, the different signal pathways among downregulation of the tight junction proteins by PE. Treatment with PE transiently downregulated mRNAs of the tight junction molecules in HNECs. These data suggest that PE rapidly induces the activation of multiple signaling mediators in HNECs and indirectly affects the synthesis of transmembrane tight junction proteins via distinct signaling pathways.

PE disables PAR-2 in A549 airway epithelial cells and in 16 HBE cells [1]. The activation of PAR-2 initiates multiple effects including enhanced airway inflammation and reactivity [13]. PAR-2 also affects the airway epithelial barrier [16]. In the present study, PE transiently reduced PAR-2 at mRNA and protein level in HNECs. Knockdown of PAR-2 using siRNA resulted in the downregulation of occludin and claudin-1 at the mRNA and protein levels. Furthermore, the knockdown of PAR-2 greatly enhanced the downregulation of occludin and claudin-1 by treatment with PE. It is thought that PAR-2 may play a crucial role in maintenance of tight junctions in HNECs. These data indicate that PE affects expression of tight junction proteins via PAR-2 in HNECs. We investigated whether PAR-2 agonist prevents the reduction of transmembrane tight junction proteins by treatment with PE in HNECs. Treatment with more than 100 μM PAR-2 agonist could prevent delocalization of occludin and claudin-1 and downregulation of the mRNAs. However, in the present study, the knockdown of PAR-2 with siRNA did not affect the barrier function in the control HNECs (data not shown). Furthermore, when we measured TER in HNECs pretreated with a PAR-2 agonist before treatment with PE, a PAR-2 agonist did not protect the disruption of barrier function by PE (data not shown). These suggest that PAR-2 in part regulates the expression of tight junction proteins but not the barrier function in HNECs.

In conclusion, PE transiently disrupts tight junctions in HNECs through multiple effects: direct degradation, distinct signal transduction, and downregulation of PAR-2. *P. aeruginosa* is related to prolonged CRS [3]. The transient disruption of tight junctions may be repeatedly caused during CRS by PE and induce secondary infection by bacteria. PAR-2 agonists might be useful for the prevention and treatment of CRS.

## Competing interests

The authors declare that they have no competing interest.

## Authors’ contributions

KN and TK carried out the genetic cell biological studies and drafted the manuscript. KO, RM and SH participated in cell culture. KT participated in the design of the study and performed the statistical analysis. TH and SN conceived of the study, and participated in its design and coordination and helped to draft the manuscript. All authors read and approved the final manuscript.

## Supplementary Material

Additional file 1 **(A) Western blotting for tight junction and adherens junction proteins in hTERT-transfected HNECs after treatment with 0.01 U neutrophil elastase.** (B) RT-PCR for mRNAs of tight junction molecules in hTERT-transfected HNECs after treatment with 0.01 U neutrophil elastase. NE: neutrophil elastase. Click here for file

Additional file 2 **Western blotting for tight junction proteins in hTERT-transfected HNECs treatment with pan-PKC inhibitor (GF109203X), MEK1/2 inhibitor (U0126), PI3K inhibitor (LY294002), p38 MAPK inhibitor (SB203580), JNK inhibitor (SP600125), epidermal growth factor (EGF) receptor inhibitor (AG1478), COX1 inhibitor (FR122047), and COX2 inhibitor, NF-κB inhibitor (IMD-0354), and Proteasome inhibitor (MG132) without** ***Pseudomonas aeruginosa *** **elastase.** Click here for file
