# Supplementary figures and images for: Pseudomonas aeruginosa elastase causes transient disruption of tight junctions and downregulation of PAR-2 in human nasal epithelial cells
Source: Respir Res. 2014 Feb 18;15(1):21. doi: 10.1186/1465-9921-15-21 (PMC3936699; doi:10.1186/1465-9921-15-21)

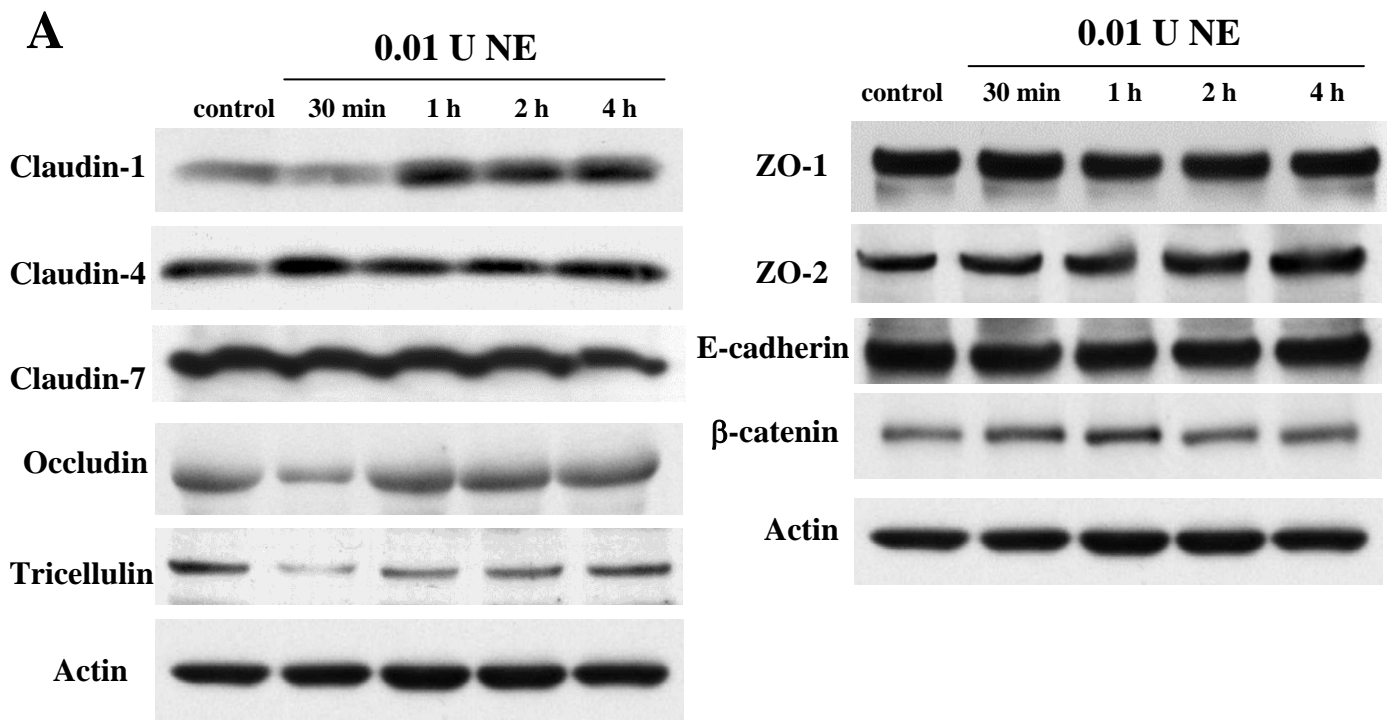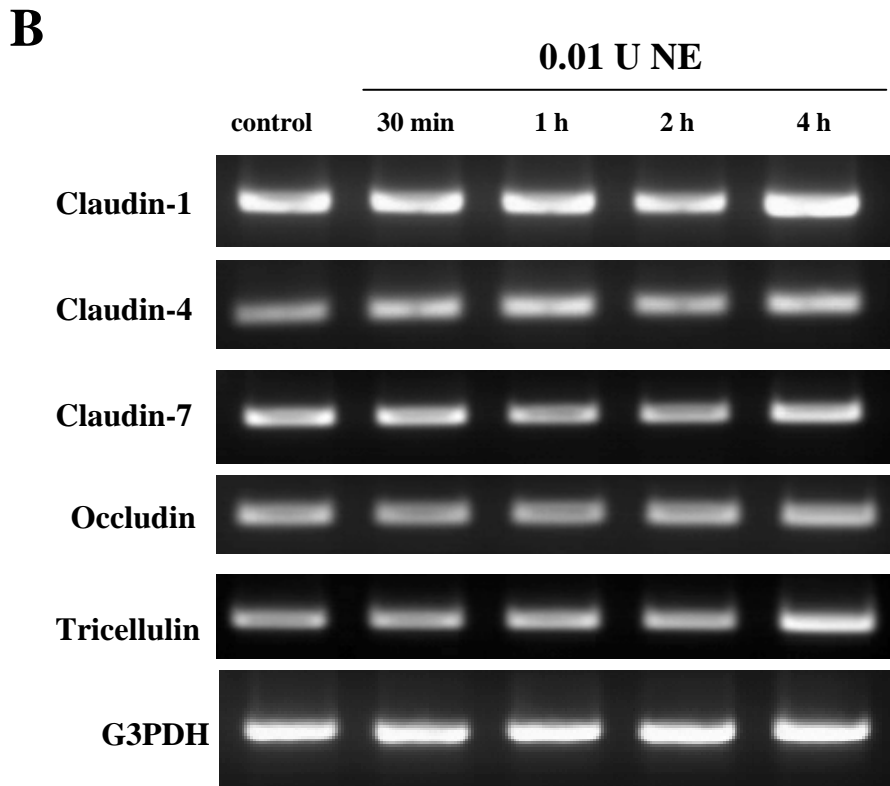

Supplement: Additional file 1 — (A) Western blotting for tight junction and adherens junction proteins in hTERT-transfected HNECs after treatment with 0.01 U neutrophil elastase. (B) RT-PCR for mRNAs of tight junction molecules in hTERT-transfected HNECs after treatment with 0.01 U neutrophil elastase. NE: neutrophil elastase. [file 1465-9921-15-21-S1.pdf]

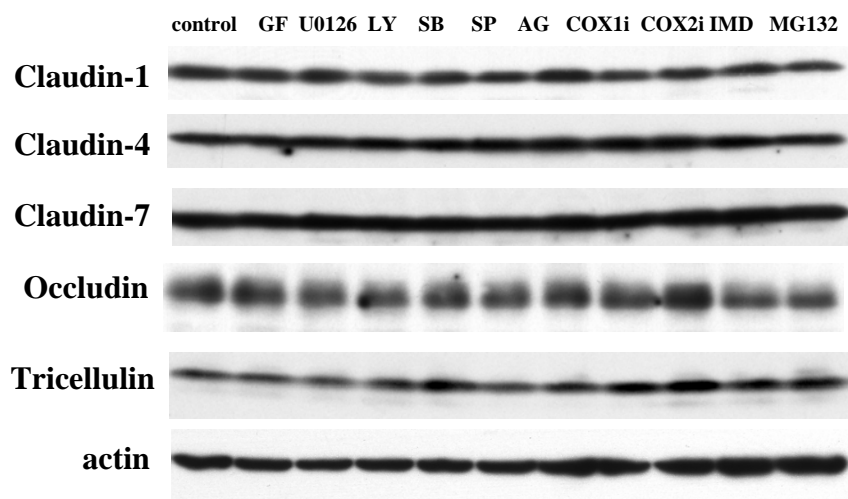

Supplement: Additional file 2 — Western blotting for tight junction proteins in hTERT-transfected HNECs treatment with pan-PKC inhibitor (GF109203X), MEK1/2 inhibitor (U0126), PI3K inhibitor (LY294002), p38 MAPK inhibitor (SB203580), JNK inhibitor (SP600125), epidermal growth factor (EGF) receptor inhibitor (AG1478), COX1 inhibitor (FR122047), and COX2 inhibitor, NF-κB inhibitor (IMD-0354), and Proteasome inhibitor (MG132) without Pseudomonas aeruginosa elastase. [file 1465-9921-15-21-S2.pdf]
